# Flavonoids: Separation and Quantitation

**DOI:** 10.1155/2015/874148

**Published:** 2015-03-01

**Authors:** Wanchai De-Eknamkul, Kaoru Umehara, Jan Frederik Stevens

**Affiliations:** ^1^Faculty of Pharmaceutical Sciences, Chulalongkorn University, Bangkok 10330, Thailand; ^2^School of Pharmaceutical Sciences, University of Shizuoka, Shizuoka 422-8526, Japan; ^3^Department of Pharmaceutical Sciences, College of Pharmacy, Oregon State University, Corvallis, OR 97331, USA

Flavonoids, products of secondary metabolism, are widely distributed in the plant kingdom. They belong to the large group of plant polyphenols and represent one of the most abundant classes of phytochemicals that are ubiquitously present in fruits, vegetables, medicinal plants, and their products. Thousands of flavonoids, existing both in free form and as glycosides, have been characterized and reported in the literature. Many of them are known to play ecological roles in the interactions of plants with their environment. Some flavonoids are known to act as signaling molecules and others as defense agents to fend off herbivores.

In addition, flavonoids are renowned for their numerous health benefits. Scientific evidence supports epidemiological observations that regular intake of dietary flavonoids reduces the risk of oxidative-stress mediated pathogenesis of human diseases as well as of age-related ailments. In many cases, however, it is necessary to release the flavonoids from plant materials by extraction before they can be evaluated for biological and pharmacological activity. For further development of health products or herbal drugs, plant extracts are usually characterized by chromatographic means for quality control purposes or subjected to separation to obtain the bioactive flavonoid constituents.

The increasing interest in bioactive flavonoids from an ecological or health perspective has resulted in the isolation and structure elucidation of many novel and minor natural flavonoids with interesting biological activities. At the same time, several techniques of chromatography have been further developed for efficient separation, identification, and quantitation of the flavonoids.

In this special issue, the contributions consist of ten papers covering several aspects of flavonoids analysis, ranging from methods and techniques of extraction, separation, and quantitation to phytochemical profiling and identification of bioactive flavonoids.

For the optimization of extraction, a chemometric approach based on response surface methodology was used in two studies. One is for the optimization of ionic liquid-based simultaneous ultrasonic and microwave-assisted extraction for isolating rutin and quercetin from leaves of* Abutilon theophrasti* (C. Zhao et al.) and the other for the optimization of reflux conditions for total flavonoid and total phenolic extraction and enhanced antioxidant capacity in* Pandanus amaryllifolius* (A. Ghasemzadeh and H. Z. E. Jaafar). A similar microwave-assisted method was used for the simultaneous extraction of luteolin and apigenin from* Paeonia ostii *pods (H. Wang et al.).

For the separation and quantitation of flavonoids, in addition to the generally used HPLC-UV, the technique of HPLC-MS/MS is exemplified in two papers: “Flavonoids in* Juglans regia* L. Leaves and Evaluation of* In Vitro* Antioxidant Activity via Intracellular and Chemical Methods” (M.-H. Zhao et al.) and “Chemical Characterization of Fruit Wine Made from Oblacinska Sour Cherry” (M. Pantelić et al.). The latter also used the powerful UHPLC system for separation of the constituents. Furthermore, the technique of HPTLC was shown to obtain good separation of the group flavone C-glycosides as reported in the paper entitled “Analysis of Flavone C-Glycosides in the Leaves of* Clinacanthus nutans* (Burm. f.) Lindau by HPTLC and HPLC-UV/DAD” by J. L. Chelyn et al.

Interestingly, the paper “Phytochemical Profiles and Antioxidant and Antimicrobial Activities of the Leaves of* Zanthoxylum bungeanum*” by Y. Zhang et al. showed very nicely the simultaneous separation of 12 flavonoids and related compounds in a single HPLC run to obtain phytochemical profiles of the plant material. Equally impressive is the paper “Phenolic Composition and Antioxidant Activity of* Malus domestica* Leaves” (M. Liaudanskas et al.) which showed complete separation of all 13 flavonoids and phenolic present in apple leaf extracts. Similarly, nine closely related flavones differing from each other by variation in ring substitution were also successfully separated as demonstrated in the report “HPLC-Fingerprints and Antioxidant Constituents of* Phyla nodiflora*” by F.-J. Lin et al.

Finally, there is one paper reporting on the isolation and structure elucidation of the flavonoids afzelin and isoquercitrin from* Ocotea notata *(I. F. B. Costa et al.). Both flavonoids strongly exhibited antimycobacterial activity and inhibition of nitric oxide production by macrophages.

We hope that all the interesting results published in this special issue will stimulate the continuing efforts to develop superior methods of flavonoid separation and quantitative analysis.

